# Impact of stroke on health-related quality of life in diverse cultures: the Berlin-Ibadan multicenter international study

**DOI:** 10.1186/1477-7525-9-81

**Published:** 2011-09-27

**Authors:** Mayowa O Owolabi

**Affiliations:** 1 Neurology Unit, Department of Medicine, University College Hospital, Ibadan, Nigeria; 2 World Federation for Neurorehabilitation, WFNR-Blossom Specialist Medical Center, First Center for NeuroRehabilitation in East, West and Central Africa, PO Box 30946, Secretariat Post Office, 200001 Ibadan, Nigeria

**Keywords:** stroke, quality of life, rehabilitation, HRQOLISP, seed of life model, spiritual, transnational, multicultural, HRQOL

## Abstract

**Background:**

Various studies have reported discordant profiles of health-related quality of life (HRQOL) after stroke. The aims of this study, the first of its kind, were to determine the real impact of stroke on HRQOL across diverse cultures; and to compare HRQOL between stroke patients and healthy adults, and across stroke severity strata.

**Methods:**

100 stroke patients and 100 apparently healthy adults (AHAs) in Nigeria; as well as 103 stroke and 50 AHAs in Germany participated. Stroke severity was measured using the National Institute of Health Stroke Scale, Stroke Levity Scale and modified Rankin scale. HRQOL was evaluated using the HRQOL In Stroke Patients (HRQOLISP) measure, a holistic multiculturally-validated measure with seven therapeutically-relevant domains distributed into two spheres.

**Results:**

Domains within the spiritual sphere were considered more important by stroke patients. In both countries, stroke patients significantly (0.00001 < p < 0.004) had worse HRQOL than AHAs in all domains within the physical sphere. This was not so for the spiritual sphere. Consistently, stroke severity correlated significantly with all domains in the physical sphere unlike the spiritual sphere. In diverse cultures, the correlation coefficients between HRQOL and all indices of stroke severity revealed a decremental trend from the physical domain (rho = 0.77, p < 0.00001) to the spiritual domain (rho = 0.01, p = 0.893).

**Conclusions:**

Consistently, stroke elicited a decremental response across domains, with domains in the spiritual sphere being relatively stroke-resilient. The potential utility of the relatively preserved spiritual sphere in facilitating stroke rehabilitation requires evaluation in diverse cultures.

## Background

Stroke, a leading cause of disability [1], is usually a major life event. The ultimate goal of stroke interventions is to improve the health-related quality of life (HRQOL) of survivors ensuring that they are enabled to fulfil their roles and purpose in life after the event. Therefore, it is imperative to know the real impact of stroke on HRQOL as a basis for planning and evaluating therapeutic and rehabilitative interventions after stroke [1].

Enormous variations have been reported in the profile of HRQOL in stroke patients [1]. Furthermore, there are conflicting reports on the relative impact of stroke on different domains of HRQOL. While some studies reported impairment of all domains even in those deemed to have recovered, other studies discordantly reported sparing of the domain assessing physical functioning or psychological functioning or autonomy [2-4].

Thus, the true impact of stroke on global and dimensional HRQOL remains unknown. This inconsistency is most probably due to considerable variations in the rigor of the methods used and the inadequacies of both qualitative and quantitative HRQOL assessment measures [1]. None of the HRQOL measures previously employed (both generic and stroke-specific) fully operationalised the concept of HRQOL [5] to embrace relevant spiritual dimensions in the stroke patients [6,7]. This is because the measures and their inherent domains, were not primarily developed on a theory of HRQOL tapping an integrative philosophy of human life. Derivation of domains solely by statistical procedures (searching for explanatory factors) without serious theoretical justification does not in itself guarantee meaningfulness and therapeutic relevance [8-10]. It can therefore be misleading to investigate HRQOL in stroke without an integrative approach [6,7]. Empirical and theoretical interpretations of the stroke experience are likely to be more realistic when dynamically incorporating both the physical and the spiritual spheres. However, many HRQOL measures do not include spiritual wellbeing as a component of HRQOL and thus neglect this core aspect of HRQOL.

Furthermore, very few of the previous studies compared HRQOL in stroke patients with healthy controls while there is no international study using the same protocol and instrument to unravel the consistent impact of stroke on HRQOL across cultures. While some of the reported wide variations in post-stroke HRQOL may be due to cultural differences, it is pertinent to ascertain which aspects of post-stroke HRQOL are stable or consistently impaired across cultures. This necessitates the cross-cultural comparison of HRQOL.

The aims of this study were to assess the impact of stroke on HRQOL in diverse cultures using a holistic measure (the HRQOL In Stroke Patients [HRQOLISP] questionnaire); and to compare HRQOL between stroke patients and healthy adults and across stroke severity strata. The HRQOLISP is a holistic multiculturally-validated measure based on an integrative concept of human life: the seed of life model (SOLM). The SOLM was derived from extensive literature research, multidisciplinary consultations, and discussion with stroke patients [8,9,11]. It was based on extensive exploration of the (often neglected) belief systems of stroke patients and reinforced by analysis of the philosophies of Socrates, Plato, Aristotle, Descartes, Spinoza and Leibniz [8,9,12,13].

**Figure 1:**
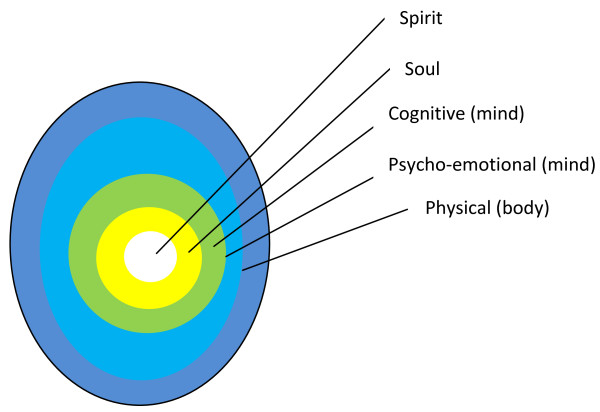
**The seed of life model (SOLM)**. The SOLM proposes a dualistic configuration of the human nature comprising the physical and spiritual spheres The egg-like model shows the relationship of different domains of quality of life as onion-like concentric zones within two spheres. The physical sphere (peripheral) includes the physical (body), psycho-emotional (mind) cognitive domains (mind) domains while the spiritual sphere (central) includes the soul and spirit domains.

The SOLM [8,9,11] is an advancement over previous theories describing the nature of human beings. Examples of earlier theories are Hartmann's and Scheller's descriptions. Whereas 'Hartmann distinguished different strata that constituted body, mind and spirit in a hierarchical pattern with the spirit at the top of the other two, Scheler distinguished three layers, the spirit being the centre and the other two layers around it ' [14]. The SOLM [8,9,12,13] proposes a combined hierarchical and concentric model, recognising a spirit domain within and above the soul domain, both of which are on top and within the other two layers [8,9,11,13]. This is graphically elaborated in Figure 1.

## Methods

### Design and Participants

The study was conducted between 2002 and 2004 at the University College Hospital, in Ibadan a major city in Nigeria; and from 2004 to 2005 at the Median Klinik, and the Evangelisches Geriatrisches Zentrum in Berlin, a major city in Germany. Nigeria is a low-income African country while Germany is a high-income European country. Ethical approvals were obtained from the ethical committees of the University of Ibadan/University College Hospital, Ibadan and Charité Universitätsmedizin, Berlin.

A multicenter international design was employed with stroke as the exposure variable and HRQOL as the outcome variable. Hospital-based medical records of stroke patients were reviewed to obtain retrospective data about stroke.

Self-reported medical histories of adult volunteers were reviewed to ascertain that they were healthy (i.e. no physical or mental illness). In Ibadan, healthy clients of the geriatric clinic who visited the clinic regularly for medical screening were recruited. In Berlin, healthy hospital workers and clients of the Sport Gesundheit Park were included. Thus, a reference group of apparently healthy adults (AHAs) [3,15] with comparable age and gender was established in each country. They provided the normative scores for the different HRQOL domains and spheres against which the degree of reduction in HRQOL by stroke could be quantified.

Consecutive stroke patients encountered within the study period who fulfilled the inclusion criteria and gave consent were included. To improve the generalizability of the findings and because the impact of stroke on HRQOL may persist for life [16] (even in those deemed to have recovered) [3,17,18], stroke patients encountered ≥ one month after stroke were included without excluding those with long post-stroke duration. Post-stroke duration was calculated based on the first-ever stroke.

Acute stroke patients were excluded because they might be clinically unstable and have communication difficulties. Others excluded from the study were patients with significant comorbidities that were not related to stroke, those with communication problems who had no reliable proxies and those who did not give consent. Proxies were considered to be reliable if they were intimate relatives of the patient who were living with him/her. The few patients who had reliable proxies were not excluded in order to avoid selection bias against those with severe stroke for whom HRQOL assessment is particularly crucial [3,19].

### Measures

HRQOL was evaluated with a valid, reliable and holistic patient-centred stroke-specific questionnaire, the 102-item HRQOLISP measure [11].

The HRQOLISP (Additional file 1- English version and Additional file 2-HRQOLISP German version) comprises 102 items. Like other HRQOL measures, these items were distributed into domains. The seven HRQOLISP domains have been validated in stroke patients and healthy individuals in whom they demonstrated good face, content, 'known groups' and construct validity as well as internal consistency and test-retest reliability [11].

Whereas other HRQOL measures have only one domain assessing spiritual functioning, the HRQOLISP has three distinct domains assessing spiritual functioning. Thus, based on their construct validity, internal consistency reliability and factorial validity, the HRQOLISP's domains were further grouped into two 'spheres' (using Hartmann's terminology [20]): 'physical' and 'spiritual'. Domains in each sphere had similar constructs as was validated by their pattern of correlation to measures of stroke severity [11].

Moreover Principal Component Analysis of the seven domains showed two principal components which explained 79% of the total variance. Component 1 (physical sphere) had an Eigenvalue of 4.42, while component 2 (spiritual sphere) had an Eigenvalue of 1.14.

Furthermore, internal consistency reliability and single factor analysis of the spheres yielded the following results. The spiritual sphere [11] (consisting of the soul, spiritual and spiritual interaction domains) had a Cronbach's alpha of 0.707 (fulfilling the Nunnaly's criterion), an Eigenvalue of 1.987 and 66.3% explanation of variance by a single factor solution. Similarly, the 'physical' sphere (operationally defined as comprising physical, psycho-emotional, cognitive and eco-social domains) had a (within-sphere inter-domain internal consistency reliability) Cronbach's alpha of 0.868 suggesting a single explanatory factor. Within the physical sphere [11], explanatory factor analysis showed that a single factor explained 74.1% of the variance with an Eigenvalue of 2.968.

In a nutshell, the domains were grouped into two spheres based on their construct validity, internal consistency reliability and factorial validity. The grouping of items of HRQOL measures into domains makes analysis and interpretation easier. Similarly, this grouping of domains into spheres, while still recognising the uniqueness of each domain, facilitates characterization and description of domains that behave alike psychometrically in contrast to other domains.

The *physical sphere *of the HRQOLISP comprises the physical domain which assesses motor, sensory and sphincteric dysfunction; the psycho-emotional domain which measures mood disorders, the cognitive domain which assesses disorders of reasoning and executive functioning; and the eco-social domain measures interpersonal and ecological interactions of the physical sphere (Additional file 1). The *spiritual sphere *comprises the 'soul' domain including items assessing self-determination, self-esteem, personal growth and autonomy [8,9,11-13]; the spiritual domain which assesses the transcendental and idealistic aspects of human life, including the individual's perceptions of the supreme meaning and purpose of life after stroke; and the spiritual interactional domain which measures interactions of the spiritual sphere (eg interactions with people of the same faith) [8,9,11-13,21]. The items within each domain are listed in Additional file 1. Thus, the HRQOLISP operationalises the concept of HRQOL as a holistic, multidimensional, subjective and patient-centered outcome measure.5 This concept is based on the WHO definition of HRQOL [22].

The HRQOLISP scores for each domain are generated by the Likert's method, i.e. item responses are summed without weighting or standardization [11]. This is done after recalibrating the items such that a high score always indicate better quality of life [11,23]. This method facilitates interpretation and inter-individual comparisons [23]. The domain scores are then transformed into a scale with a maximum score of 100 (best health) each. The score for each sphere is generated by averaging the scores of the constituent domains [11]. Similarly, the total HRQOLISP score is generated by finding the arithmetic mean of all domain scores [11].

Stroke severity was evaluated with the National Institute of Health Stroke Scale (NIHSS) and Stroke levity scale (SLS). The SLS correlates significantly to the NIHSS (rho = -0.79, p < 0.0001) and can be applied in illiterate populations [24]. The modified Rankin scale (mRS) was used to measure disability. The NIHSS, SLS, and mRS were applied by the investigator to the patients in their respective hospitals.

The HRQOLISP was applied to consecutive patients or their reliable proxies. To ensure honest responses to personal questions, the preferred mode of administration was self-completion by the respondents. However, if the patient or proxy was unable to read and write, it was applied by face-to-face interview conducted by the same investigator in both countries. To assess the effect of mode of administration on responses, a subset of five respondents had the questionnaire administered to them by the interviewer after they had completed the questionnaire by themselves.

The hypothesis tested in the data analysis was that 'despite cultural and religious differences, patients suffering from stroke, which is primarily a physical ailment, would have their spiritual functioning preserved relative to their physical functioning.' Specifically, 'across diverse cultures, the severity of stroke should correlate significantly with domains measuring the physical aspects of quality of life rather than domains assessing the spiritual components of quality of life.'

### Statistical analysis

Socio-demographic data collected from the patients, including age, gender and occupation, were collated and summarized. Differences between stroke patients and AHAs were analyzed using student's t-test for continuous variables and chi-square for categorical variables. HRQOLISP and SLS scores were generated with previously described methods [24]. HRQOLISP scores were compared between stroke patients and AHAs in both cities using student's t test and ANCOVA controlling for differences in socio-demographic variables (gender, level of education, and occupational strata). Mean differences between stroke patients and AHAs were obtained for the physical and spiritual spheres. Spearman ranks correlation statistics was used to explore relationships between stroke severity and the different domains of HRQOLISP. A p value of < 0.05 was taken to be significant. Statistical analyses were conducted using the SPSS software.

## Results

The socio-demographic and clinical characteristics of the participants are summarized in Table 1 for both cities. A total of 353 respondents [100 stroke patients and 100 apparently healthy adults (AHAs) in Ibadan; and 103 stroke and 50 AHAss in Berlin] were assessed. Those excluded from the study were patients with significant comorbidities that were not related to stroke (n = 4 in Ibadan, n = 5 in Berlin), those with communication problems who had no reliable proxies (n = 4 in Berlin) and those who did not give consent (n = 6 in Berlin). Of the Ibadan stroke patients 88% were Yoruba, 4% were Igbo, and 2% were Hausa; 69% were Christians while 31% were Muslims. 100% believed in God while 94% believed strongly in life after death. In Berlin, 89% of the stroke patients were Germans, 3% were Turkish; 65% were Christians while 5% were Muslims. There was one Buddhist while the remainder had no religious affiliation. 63% of the stroke patients believed in God while 37% believed in life after death.

**Table 1:** Sociodemographic and clinical characteristics

	IBADAN			BERLIN		
**Variable**	**Stroke patients n(%) = 100**	AHAs* n(%) = 100	**Tests of significance**	**Stroke patients n = 103**	AHAs* n = 50	**Tests of significance**

**Age, yrs**						
Mean (SD)	59.4(9.9)	57.6 (12.4)	t = 1.138, 95% CI -1.319 to 4.919, p = 0.256	66.9 (11.6)	65.7(5.9)	t = 0.676, 95% CI -2.258 to 4.606,
30- 49	15	27		12	1	p = 0.500
50- 69	71	48		42	39	
70- 99	14	25		49	10	
**Gender**						
Male	41	41	identical	61	11	*χ^2^*= 18.720 p <0.0001
Female	59	59		42	39	
**Occupation**						
Skilled/Semi-skilled Workers	44	72		33	7	
Unskilled Workers	51	27		2	0	
Pensioner	0	0		68	43	
Others	5	1	*χ^2^*= 18.06, p = 0.021	0	0	*χ^2^*= 9.042 p = 0.171
**Education**						
None	35	21		3	0	
Primary (1-6 yr)	15	10		39	10	
Secondary (7- 12 yr)	30	19	*χ^2 ^= 19.4, p = 0.001*	31	23	*χ^2^ = 7.483, p = 0.058 *
Tertiary (> 12 yr)	20	50		30	17	

**Stroke type**	(clinical*, CT)			(CT/MRI)		
Ischemic	30 63			80		
Hemorrhagic	23 37			11		
Indeterminate/Mixed	47			9 (mixed)		
**Recurrent stroke**	16			22		
**Time since first stroke**						
Median, Range (months)	28.5, 1 to 348			1.5, 1 to 324		
**Modified Rankin Scale**						
No symptom/sign. disability	16			4		
Slight disability	27			37		
Moderate disability	24			24		
Moderately severe disability	31			8		
Severe disability	2			27		
**Stroke levity score**						
0-5 (severe impairment)	6			6		
6-10 (mod. impairment)	14			26		
11-15 (mild impairment)	80			68		
**NIHSS**						
0-5				64		
6-10				19		
11-16				17		

AHAs*: Apparently healthy adults. Using the WHO definition of stroke, the clinical distinction of stroke from other disorders has a sensitivity of up to 95% and a specificity of up to 97%, while the classification of stroke subtypes using the WHO stroke scales have a sensitivity of up to 68% and specificity of 67% and is better when assessment is by a neurologist (as was done in this study). *Ogun SA, Oluwole O, Ogunsehinde O, Fatade B, Odusote KA: Misdiagnosis of stroke -a computerized tomography study. West Afr J Med 2000;20:19-22*.

Analysis of relevant items in the SLS and HRQOLISP revealed aphasia in 31% in Ibadan and 38% in Berlin; sexual dysfunction in 45% in Ibadan and 80% in Berlin; and post stroke emotional disorder in 75% in Ibadan and 68% in Berlin.

In the subset of five respondents who had the questionnaire administered to them by two methods, there was strong correlation between the HRQOL scores obtained by interview and self-administration (0.96 < r < 0.99, 0.000001 < p < 0.036).

**Figure 2:**
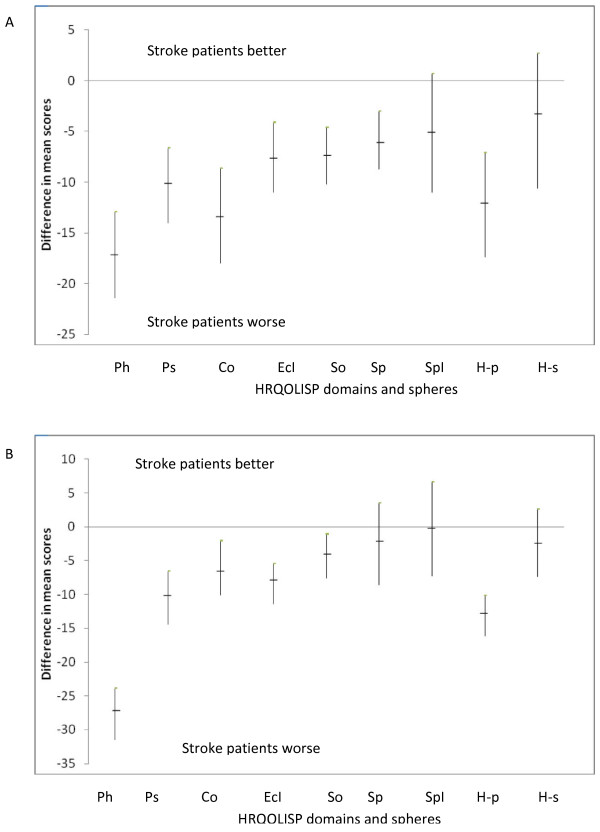
**Difference between mean HRQOLISP scores for stroke patients and apparently healthy adults.** A: Difference between mean HRQOLISP scores for stroke patients and apparently healthy adults (Ibadan). Ph: Physical domain, Ps: Psycho-emotional domain, Co: Cognitive domain, EcI: Ecosocial Interaction domain, So: Soul domain, Sp: Spirit domain, SpI: Spiritual Interaction domain, H-p: HRQOLISP physical sphere, H-s: HRQOLISP spiritual sphere. B: Difference between mean HRQOLISP scores for stroke patients and apparently healthy adults (Berlin). Ph: Physical domain, Ps: Psycho-emotional domain, Co: Cognitive domain, EcI: Ecosocial Interaction domain, So: Soul domain, Sp: Spirit domain, SpI: Spiritual Interaction domain, H-p: HRQOLISP physical sphere, H-s: HRQOLISP spiritual sphere.

In both Ibadan and Berlin, all domains were rated at least moderatelyimportant by AHAs and stroke patients. Domains in the spiritual sphere were accorded higher importance rating by stroke patients than by AHAs in both cities. The mean HRQOLISP scores for the AHAs were similar in the physical sphere in Berlin and Ibadan, but higher in the spiritual sphere in Ibadan than Berlin (Tables 2 and 3). Compared to AHAs, HRQOL was worse in stroke patients in both cities in all domains (Figures 2A and 2B). After controlling for possible confounders (age, gender, socioeconomic class), there was significant difference between AHAs and stroke patients in every domain in the physical sphere in both cities (0.006 < p < 0.00001, Tables 2 and 3). This was not so in the spiritual sphere. The mean difference in HRQOL between AHAs and stroke patients was much greater in the physical sphere than the spiritual sphere in both cities (Tables 2 and 3, Figures 2A, and 2B).

**Table 2:** HRQOL Profile in Stroke patients and Apparently healthy adults (AHAs) -Ibadan

Domains	Stroke patients Mean (SD)	AHA^s Mean (SD)	Mean difference, (95%CI)	*t*	*p *(two-tailed)	*F ANCOVA* *(adjusted for age and SEC†)*	*p (adjusted for age and SEC†)*
**Physical Sphere**							
Physical	73.9 (14.1)	91.1 (7.0)	-17.2 (-21.4, -12.9)	-7.937	< 0.00001*	9.953	< 0.00001*
Psycho-emotional	74.4 (13.5)	84.7 (8.8)	-10.3 (-14.0,-6.6)	-5.553	< 0.00001*	5.345	0.002*
Cognitive	71.9 (13.1)	85.0 (17.0)	-13.1 (-18.0, -8.6)	-5.481	< 0.00001*	8.461	< 0.00001*
*Ecosocial Interaction*	69.9 (12.7)	76.8 (10.4)	-6.9 (-11.0, -4.1)	-3.430	0.001*	6.620	< 0.00001*
**Spiritual sphere**							
Soul	76.8 (6.9)	84.2 (6.0)	-7.4 (-10.2, -4.6)	-5.179	< 0.00001*	7.281	< 0.00001*
Spirit	78.9 (10.8)	84.8 (9.2)	-5.9 (-8.7, -3.0)	-4.028	< 0.00001*	4.763	0.003*
*Spiritual interaction*	76.8 (13.0)	82.0 (26.2)	-5.2 (-11.0, 0.7)	-1.726	0.087	1.454	0.230
**HRQOLphysical sphere**	71.4 (10.2)	83.6 (6.7)	-12.2 (-17.4, -7.1)	-4.763	< 0.00001*	7.031	0.001*
**HRQOLspiritual sphere**	76.5 (8.2)	83.7 (7.4)	-7.2(-10.6,-3.6)	-4.030	< 0.001*	3.757	0.016*
HRQOLsum	73.5 (9.1)	84.4 (6.9)	-10.9 (-17.0, -4.8)	-3.496	0.002*	3.883	0.027*

AHAs^: Apparently healthy adults. SEC†: Socioeconomic class (Socioeconomic class is based on level of education, occupational strata and average monthly income). * Statistically significant

**Table 3:** HRQOL Profile in Stroke patients and Apparently healthy adults (AHAs)-Berlin

Domains	Stroke Patients Mean (SD)	AHAs^ Mean (SD)	Mean difference (95% confidence interval)	*t*-value	*p*	*F ANCOVA (adjusted for age, sex and SEC†)*	*p (adjusted for age, sex and SEC†)*
**Physical sphere**							
Physical	65.1 (13.0)	92.7 (5.1)	-27.6 (-31.4, -23.8)	-14.365	< 0.00001*	73.96	< 0.00001*
Psycho-emotional	74.1 (12.3)	84.6 (9.6)	-10.5(-14.4, -6.5)	-5.237	< 0.00001*	10.163	< 0.00001*
Cognitive	75.5 (13.0)	81.5 (8.9)	-6.0 (-10.1, -2.0)	-2.927	0.004*	4.328	0.006*
*Ecosocial Interaction*	68.3 (9.1)	76.8 (7.9)	-8.5 (-11.4, -5.4)	-5.835	< 0.00001*	20.481	< 0.00001*
**Spiritual sphere**							
Soul	65.4 (9.7)	69.7 (9.1)	-4.3 (-7.6, -1.0)	-2.645	0.009*	2.460	0.065
Spirit	46.6 (18.3)	49.1 (17.5)	-2.5 (-8.6, 3.6)	-0.817	0.416	0.912	0.437
*Spiritual interaction*	45.3 (22.0)	45.6 (17.6)	-0.3 (-7.3, 6.7)	-0.073	0.942	0.495	0.686
**HRQOLphysical sphere**	70.8 (9.6)	83.8 (6.3)	-13.0(-16.1, -10.1)	-8.615	< 0.000001*	21.325	< 0.00001*
**HRQOLspiritual sphere**	52.4 (15.6)	54.8 (13.3)	-2.4 (-7.4, 2.7)	-0.918	0.36	1.82	0.128
HRQOLsum	62.8 (8.9)	71.4 (7.7)	-8.6 (-11.5, -5.6)	-6.075	< 0.000001*	11.387	< 0.00001*

AHAs^: Apparently healthy adults, SEC †: Socioeconomic class (Socioeconomic class is based on level of education, occupational strata and average monthly income)

* Statistically significant.

**Figure 3:**
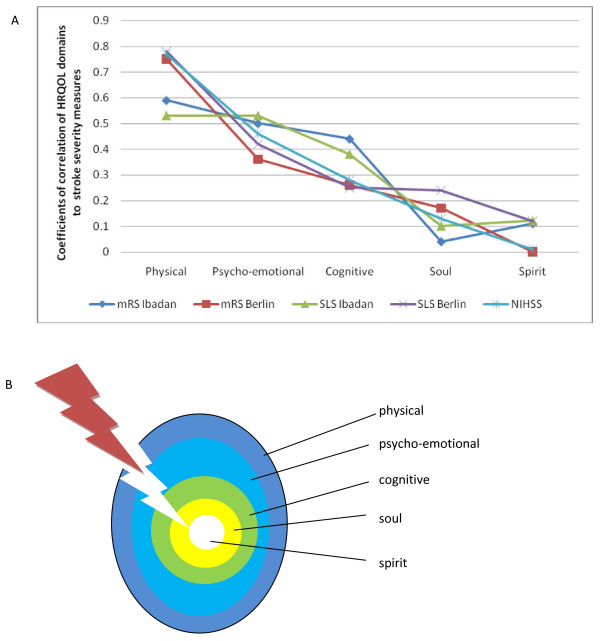
**Decremental impact of stroke across HRQOL domains. **A: Scalar plot of correlation coefficients of HRQOLISP domains with stroke severity indices. A decremental response is elicited from the physical to the spiritual domains, thus supporting the SOLM. B: The seed of life model: The egg-like model shows the relationship of different domains of quality of life as onion-like concentric zones with the physical domain outermost, thus bearing the maximal impact when stroke strikes.

In both countries, in contrast to domains within the spiritual sphere, stroke severity correlated significantly with all domains in the physical sphere (Table 4). Furthermore, examination of the correlation coefficients between HRQOL and indices of stroke severity revealed a progressive decrease from the physical (rho = 0.77, p < 0.00001) to the spiritual domain (rho = 0.01, p = 0.893, Table 4, Figures 3A and 3B).

**Table 4:** Correlation of HRQOLISP domains and spheres to measures of stroke severity in Berlin and Ibadan

HRQOLISP Domains	mRS^ Ibadan rho, p	**mRS Berlin**, rho, p	SLS‡Ibadan, rho, p	SLS Berlin, rho, p	**NIHSS† Berlin**, rho, p
**Physical sphere**					
Physical	-0.59, < 0.0001*	-0.75, < 0.0001*	0.53, 0.001*	0.78, < 0.0001*	-0.77, < 0.0001*
Psycho-emotional	-0.50, < 0.0001*	-0.36, < 0.0001*	0.53, < 0.0001*	0.42, < 0.0001*	-0.46, < 0.0001*
Cognitive	-0.44, < 0.0001*	-0.26, 0.007*	0.38, < 0.0001*	0.25, 0.012*	-0.28, 0.004*
*Ecosocial interaction*	-0.48, < 0.0001*	-0.50, < 0.0001*	0.45, < 0.0001*	0.49, < 0.0001*	-0.46, < 0.0001
**Spiritual sphere**					
Soul	-0.04, 0.812	-0.17, 0.080	0.10, 0.591	0.24, 0.013*	-0.13, 0.204
Spirit	-0.11, 0.276	0.00, 0.964	0.12, 0.270	0.12, 0.235	-0.01, 0.893
*Spiritual interaction*	-0.11, 0.267	0.03, 0.782	0.19, 0.071	0.11, 0.283	0.012, 0.904
**HRQOLphysical sphere**	-0.78, < 0.0001*	-0.56, < 0.0001*	0.72, 0.002*	0.59, < 0.0001*	-0.61, < 0.0001*
**HRQOLspiritual sphere**	-0.13, 0.458	-0.03, 0.763	0.30, 0.096	0.15, 0.124	-0.03, 0.738

* Statistically significant.

mRS^: modified Rankin Scale

SLS‡: Stroke Levity Scale

NIHSS†: National Institute of Health Stroke Scale

## Discussion

The study of HRQOL involves the assessment of multiple subjective realities in constant flux [6,7]. Although weighted individualized measures and qualitative methods are useful for in-depth understanding of impact of stroke on individuals, quantitative methods are better for describing patterns. Holistic quantitative measures capture all subjective realities which are crucial to the re-establishment of a sense of identity by the patient. The HRQOLISP used in this study, is the only example of such a measure developed for stroke. It captures all hitherto un-assessed subjective realities of stroke patients thereby demonstrating the real impact of stroke on different aspects of HRQOL.

Ideally, to measure the true impact of stroke on HRQOL, a prospective cohort of patients at high risk of stroke would be recruited and their HRQOL would be measured just before and after stroke. The difference in HRQOL so derived would be ascribed to the stroke event. However such a study which is not cost-efficient, would require the recruitment of very large number of patients which may eventually yield very few stroke patients resulting in poorly generalizable results as in the Framingham study (where only 10 stroke patients were recruited eventually) [16].

Therefore, the realistic design for measuring the impact of stroke on HRQOL is to compare the HRQOL in stroke patients with normative data from a healthy reference group. This assumes that the HRQOL of the stroke patient before the stroke is approximately the HRQOL of the healthy population.

### Profile of HRQOL in stroke patients compared to AHAs

This design revealed that stroke consistently resulted in worse HRQOL scores in all domains in both countries (Tables 2 and 3, Figures 2A and 2B). Although a few studies using measures that were not originally designed for stroke patients [11] have recorded no difference [15,16] in HRQOL between stroke patients and normative population, several studies using different measures have recorded worse performance by stroke patients in the limited number of domains assessed by them [2,4,18].

Thus, in comparison to AHAs, impairment of physical, psycho-emotional, cognitive and eco-social domains appears to be a consistent finding in stroke [2,4,18,25]. However, most studies did not go further to determine the relative severity of impairment of different domains. Where this was done, the findings were conflicting. Whereas physical health was reported to be worse than mental health in stroke patients in Auckland [3], the reverse was the case in Netherlands [4]. In both Berlin and Ibadan, within the physical sphere, stroke had the greatest impact on the physical domain. Furthermore, the magnitude of difference in HRQOL between stroke patients and AHAs was consistently higher for domains in the physical sphere than the spiritual sphere. There was a trend towards progressive decrease in this magnitude from the physical (outermost) to the spiritual (innermost) domains (Tables 2 and 3, Figures 2A, B, and 3B).

### The relative impact of stroke on HRQOL spheres

Consistently, the spiritual sphere was relatively stroke-resistant (Tables 2 and 3, Figures 2A and 2B). Therefore, stroke had a dualistic impact on HRQOL, significantly reducing HRQOL scores for the physical sphere in both countries, while relatively sparing the spiritual sphere. This phenomenon of disability disparity was not demonstrated by other studies using tools that neglected the spiritual sphere [26,27]. Nevertheless, Clarke (2002) used the Ryff's measure of psychological wellbeing, and found the preservation of the 'autonomy 'and 'purpose in life' domains despite significantly lower scores of all other domains [2]. These preserved domains of the Ryff's measure contain items similar to those in the spiritual sphere of the HRQOLISP [2,28].

Furthermore, domains in the spiritual sphere were considered more important by stroke patients. This was so, even in Berlin, where religious beliefs were less intense than in Ibadan. This high rating of the spiritual sphere is probably due to its documented pivotal role in the re-establishment of continuity of self [6,7,21,29,30] along the path to recovery, self-rediscovery and self-rejuvenation after stroke. This pathway is hypothetically guided by the inner light of sense of identity, purpose in life and self-determination (will power) which drives the processes of role and need re-prioritisation resulting in internal adaptation. This culminates in the formulation and deployment of coping strategies based on residual and restored personal resources. This hypothesis on the pathway to recovery is best tested in prospective studies conducted in diverse cultures because differences in spiritual functioning may have implications for the processes of internal adaptation in diverse settings [6,7,29,30]. For instance, despite the near-identical scores among AHAs in the physical sphere in both countries, the scores for domains in the spiritual sphere in stroke patients and AHAs in Berlin were less than in Ibadan (Tables 2 and 3). This is probably due to the difference in religious beliefs and affiliations in both countries, which may have implications for the processes of internal adaptation in both countries [6,7,29,30].

### The impact of stroke severity on HRQOL domains

In both cities, in clear contrast to the domains in the spiritual sphere, all domains in the physical sphere correlated significantly to all measures of stroke severity (Table 4). Thus, stroke severity had no significant impact on the spiritual sphere. This further confirms the observed dualistic impact of stroke on HRQOL thereby supporting the division of the HRQOLISP domains into two spheres.

Additionally, in Ibadan and Berlin, a decremental trend in the correlation coefficients of stroke severity to HRQOL was consistently demonstrated across domains going from the outermost to the innermost domain. The strongest correlation was found to the physical domain while the weakest was to the spiritual domain (Table 4, Figures 3A and 3B). This decremental response elicited by stroke is a novel finding which further supports the arrangement of the domains in the SOLM.

Taken together, these findings have implications for evidence-based rehabilitation service planning and health resource allocation (e.g., amount of specialists and services needed for rehabilitation of stroke survivors) [2,6,7,13]. For instance, the greater impact of stroke on the physical domain favours the allocation of more resources for the delivery of physical therapy.

Nevertheless, due to the documented [6,7,29,30] pivotal role of the spiritual sphere in rehabilitation, and its high importance rating by stroke patients, more research resources are needed for the development of therapeutic techniques aimed at exploiting this stroke-resistant sphere of HRQOL. This spiritual sphere could serve as a springboard for effecting internal adaptation, instituting coping strategies and rejuvenating other aspects of HRQOL [14,29,30]. A review of existing research has shown that spirituality is linked to positive physical and mental health outcomes in individuals with disabilities because it is used by many to help adjust to their impairments and to give new meaning to their lives [29,30]. In this respect, other aspects of spirituality rather than religious beliefs alone may be more important for positive adjustment to life changes [29,30].

### Strengths, limitations and future directions

This is the first study of HRQOL in stroke patients to use a holistic well-validated measure in a transnational multicultural setting comparing a low-income African country to an industrialized high-income European country. In these contrasting settings, the same protocol was applied including the establishment of normative groups of AHAs. This comparison group was well-matched for age and gender in Ibadan, and age in Berlin. The incomplete matching of the comparison group for gender in Berlin was controlled for in the analysis using ANCOVA. Furthermore, subgroup analysis comparing male stroke patients to male AHAs and female stroke patients to female AHAs yielded similar results with mean differences in HRQOL being substantially greater in the physical sphere.

The consistent observation of the dualistic impact of stroke on HRQOL and its decremental response across domains are unique and novel. Prospective multicultural transnational studies are required to explore this pattern and unravel the dynamic interplay between the physical and spiritual spheres of HRQOL. As illustrated in Figure 3B, the greater impact of stroke on the physical sphere may be due to its superficial position, which places it in the path of an external and physical assault such as stroke. However, further studies are needed to discover how and why the spiritual sphere is relatively preserved. It would also be worthwhile to study the impact of different modalities of therapies on these dual realities. Meanwhile, it should be noted that spiritual wellbeing may not be preserved in every stroke patient. Therefore, healthcare providers need to assess patients individually and holistically.

## Conclusions and implications

Consistently, in diverse cultural settings with different religious and ethnic identities, stroke had a dualistic impact on HRQOL. It elicited a decremental response across domains, with domains in the spiritual sphere being relatively stroke-resistant. While the more affected physical sphere should be the primary target for restorative therapy, the relatively preserved spiritual sphere could help to promote coping. In this respect, the preserved spiritual sphere could serve as a trigger for revitalizing other aspects of HRQOL.

In diverse cultures, therapeutic exploitation of these personal resources might facilitate adaptive processes and even promote the impact of restorative interventions for the physical sphere. However, the potential of the spiritual sphere to reduce the biographical impact of stroke is likely to be modified by its post-ictal salience in a given cultural and personal context. Prospective studies are warranted to exploit the dynamics of this novel paradigm. This may serve as a model for other chronic neurologic conditions with potential biographic impact.

## Abbreviations

HRQOL: Health related quality of life; HRQOLISP: Health related quality of life in stroke patients questionnaire; SLS: stroke levity scale; SOLM: seed of life model; NIHSS: National institute of health stroke scale; mRS: modified Rankin scale; AHA: Apparently healthy adults; SEC: socioeconomic class

## Competing interests

The author declares that they have no competing interests.

## Authors' contributions

MOO conceived and designed the study. He collected and analysed the data and drafted the manuscript.

## Author information

Dr. Mayowa Ojo OWOLABI,

MBBS, MWACP, FMCP, Dr. med.-magna cum laude (Berlin), Cert. Epid. & Glob. Health (Dundee)

Senior Lecturer and Consultant Neurologist, Department of Medicine,

University College Hospital, Ibadan. Nigeria.

Pioneering Regional Vice President (East, West and Central Africa),

*World Federation for Neurorehabilitation*.

Email address: mayowaowolabi@yahoo.com

Telephone Number: +234 802 077 5595

+234 807 849 6775

## Supplementary Material

Additional file 1 **Health-Related Quality Of Life In Stroke Patients (HRQOLISP) questionnaire-original international version**. The file contains the complete HRQOLISP instrument. Click here for file

Additional file 2 **Health-Related Quality Of Life In Stroke Patients (HRQOLISP) questionnaire-German version**. The file contains the complete German version of the HRQOLISP instrument. Click here for file
