# Supplementary material for: Impact of stroke on health-related quality of life in diverse cultures: the Berlin-Ibadan multicenter international study
Source: Health Qual Life Outcomes. 2011 Sep 27;9:81. doi: 10.1186/1477-7525-9-81 (PMC3206409; doi:10.1186/1477-7525-9-81)
Supplement: Additional file 1 — Health-Related Quality Of Life In Stroke Patients (HRQOLISP) questionnaire-original international version. The file contains the complete HRQOLISP instrument. [file 1477-7525-9-81-S1.PDF]

©Owolabi MO

THE HEALTH_RELATED QUALITY OF LIFE IN STROKE PATIENTS (HRQOLISP) QUESTIONNAIRE

INSTRUCTIONS

This assessment asks about how you perceive your current state of health, quality of life, or other areas of your life. **Please answer all the questions honestly**. If you are unsure about what response to give to a question, please choose the nearest most appropriate response. **Please keep in mind your standards, hopes, pleasures and concerns. Think about your life in the last two weeks. You should circle the number that best fits your response.**

1.1.1.1. : PHYSICAL DOMAIN (i-iii) to be completed by the investigator. **Items i to iv are for the Stroke Levity Scale.**

i	Best motor power in the dexterous hand/ upper limb	0 nil	1 flicker	2 gravity eliminated	3 against gravity	4 against resistance	5 normal
iia.	Best motor power in the affected upper limb	0	1	2	3	4	5
iib	Best motor power in the affected lower limb	0	1	2	3	4	5
iii.	Speech defect [aphasia]	nil 0	present 1’				
iv.	Mobility	bed bound 1	chair bound 2	walks with helpers 3	walks with aids[frame/ tripod] 4	walks unaided 5	

		**Not at all 1**	**A little 2**	**A moderate amount 3**	**Very much 4**	**Extremely 5**
v	To what extent do you have difficulties gripping objects, turning door-knob, using cutlery, writing, opening jar/can, carrying heavy objects?	0	1’	2’	3’	4’
vi	To what extent do you have difficulties controlling your bladder/bowels?	0	1’	2’	3’	4’
vii	To what extent do you have difficulties sitting/standing without losing your balance?	0	1’	2’	3’	4’
viii	To what extent do you have difficulties seeing objects off to one side/ reaching for objects because of poor eyesight?	0	1’	2’	3’	4’
ix	To what extent do you think physical pain/discomfort /abnormal sensation/absent sensation prevent you from doing what you need to?	0	1’	2’	3’	4’

©Owolabi MO

x	How much do you need any medical treatment [drugs or aids] and/or hospital attendance to function in your daily life?	0	1’	2’	3’	4’
xi	To what extent has your sex life been adversely affected?	0	1’	2’	3’	4’
		**Very dissatisfied 1**	**Dissatisfied 2**	**Neither satisfied nor dissatisfied 3**	**Satisfied 4**	**Very satisfied 5**
xii	How satisfied are you with your ability to perform your daily living activities [feeding, bathing, toileting, dressing, grooming, e.t.c.]?	1	2	3	4	5
xiii	How satisfied are you with your capacity for work?	1	2	3	4	5
xiv	How satisfied are you with your sex life?	1	2	3	4	5
xv	**How important to you are the aspects of your life covered in questions iv-xiv in this section?**	**Not at all 1**	**A little 2**	**Moderately 3**	**Very much 4**	**Extremely 5**

**1.1.1.2**	**EMOTION/PSYCHOLOGICAL DOMAIN**	**Not at all/ Never 1**	**A little/ Seldom 2**	**Moderately/ Quite often 3**	**Mostly/ Very often 4**	**Completely/Always 5**
**i**	How often do you have negative feelings such as blue mood, anger, despair, anxiety, depression, fear?	0	1’	2’	3’	4’
**ii**	Do you have enough energy for everyday life?	1	2	3	4	5
**iii**	To what extent are you able to accept your bodily appearance?	1	2	3	4	5
**iv**	To what extent do you enjoy your work?	1	2	3	4	5
**v**	How often do you laugh?	1	2	3	4	5
**vi**	To what extent do you enjoy recreation/pastimes/leisure/rest /relaxation?	1	2	3	4	5

©Owolabi MO

**vii**	How safe do you feel in your daily life?	1	2	3	4	5
**viii**	To what extent have you ever felt death to be better than your present condition?	0	1’	2’	3’	4’
**ix**	To what extent have you ever felt like ending your life?	0	1’	2’	3’	4’
		**Very dissatisfied 1**	**Dissatisfied 2**	**Neither satisfied nor dissatisfied 3**	**Satisfied 4**	**Very satisfied 5**
**x**	How satisfied are you with your sleep [duration and quality]?	1	2	3	4	5
**xi**	How satisfied are you with your feelings?	1	2	3	4	5
**xii**	**How important to you are the aspects of your life covered in questions i-xi in this section?**	**Not at all 1**	**A little 2**	**Moderately 3**	**Very much 4**	**Extremely 5**

**1.1.2**	**COGNITIVE DOMAIN**	**Not at all 1**	**A little 2**	**Moderately 3**	**Very much/ Mostly 4**	**Extremely /Completely 5**
**i**	How well are you able to concentrate?	1	2	3	4	5
**ii**	To what extent is your memory impaired?	0	1’	2’	3’	4’
**iii**	To what extent are you able to learn new things?	1	2	3	4	5
**iv**	To what extent do you understand your disease process?	1	2	3	4	5
**v**	To what extent are you able to think out/plan logical solutions to [your] problems and take decisions?	1	2	3	4	5
**vi**	To what extent are you able to relax your mind?	1	2	3	4	5
**vii**	How available to you is the information that you need for your day-to-day life?	1	2	3	4	5
**viii**	To what extent are you able to communicate?	1	2	3	4	5

©Owolabi MO

		Very dissatisfied 1	Dissatisfied 2	Neither satisfied nor dissatisfied 3	Satisfied 4	Very satisfied 5
**ix**	How satisfied are you with your memory and ability to concentrate?	1	2	3	4	5
**x**	How satisfied are you with your ability to communicate?	1	2	3	4	5
**xi**	How satisfied are you with your ability to think and learn?	1	2	3	4	5
**xii**	**How important to you are the aspects of your life covered in questions i-xi in this section?**	**Not at all 1**	**A little 2**	**Moderately 3**	**Very much 4**	**Extremely 5**

**1.2.1**	**SOUL DOMAIN**	**Not at all 1**	**A little 2**	**Moderately 3**	**Very Much 4**	**Extremely 5**
**i**	How much do you value yourself?	1	2	3	4	5
**ii**	How much confidence do you have in yourself?	1	2	3	4	5
**iii**	How much confidence do you have in your God?	1	2	3	4	5
**iv**	How creative are you?	1	2	3	4	5
**v**	To what extent are you independent and individualistic in reasoning and taking decisions?	1	2	3	4	5
**vi**	To what extent do you believe you have a purpose for living?	1	2	3	4	5
**vii**	To what extent are you interested in fulfilling your purpose for living?	1	2	3	4	5
**viii**	To what extent do you accept/ believe in destiny/predestination?	1	2	3	4	5
**ix**	To what extent do you believe in freewill?	1	2	3	4	5
**x**	To what extent do you think your present condition has prevented you from fulfilling your purpose of life?	0	1’	2’	3’	4’
**xi**	To what extent do you think your present condition has assisted you in fulfilling your purpose of life?	1	2	3	4	5

©Owolabi MO

**xii**	To what extent have your dreams/visions/ESP [if any]portrayed your present condition bad?	0	1’	2’	3’	4’
**xiii**	To what extent are you intuitive/ inspired/ ingenuous?	1	2	3	4	5
**xiv**	To what extent do you rely on God to solve your problems?	1	2	3	4	5
**xv**	To what extent do you rely on yourself to solve your problems?	1	2	3	4	5
**xvi**	To what extent do you believe the devil is responsible for your present situation?	0	1’	2’	3’	4’
**xvii**	To what extent are you or other people responsible for your situation more than God?	0	1’	2’	3’	4’
**xviii**	To what extent do you believe in afterlife?	1	2	3	4	5
**xix**	To what extent do you believe in God?	1	2	3	4	5
**xx**	To what extent do you practice your religion/faith?	1	2	3	4	5
**xxi**	To what extent do you accept your present state?	1	2	3	4	5
**xxii**	How strong is your will to live?	1	2	3	4	5
		**Very dissatisfied 1**	**Dissatisfied 2**	**Neither satisfied nor dissatisfied 3**	**Satisfied 4**	**Very satisfied 5**
**xxiii**	To what extent are you satisfied with your faith in God?	1	2	3	4	5
**xxiv**	How satisfied are you with yourself?	1	2	3	4	5
**xxv**	How satisfied are you with your abilities?	1	2	3	4	5
**xxvi**	**How important to you are the aspects of your life covered in questions i-xxv in this section?**	**Not at all 1**	**A little 2**	**Moderately 3**	**Very much 4**	**Extremely 5**

©Owolabi MO

1.2.2	**SPIRIT DOMAIN**	**Not at all 1**	**A little 2**	**Moderately 3**	**Very much 4**	**Extremely 5**
**I**	To what extent do you understand God?	1	2	3	4	5
**Ii**	To what extent are you guided / motivated by God in your [daily] life?	1	2	3	4	5
**Iii**	To what extent do you understand your religion/faith?	1	2	3	4	5
**Vi**	To what extent do you think God is responsible for your present state?	1	2	3	4	5
**V**	To what extent do you perceive your life to be meaningful?	1	2	3	4	5
		**Very Dissatisfied 1**	**Dissatisfied 2**	**Neither satisfied nor dissatisfied 3**	**Satisfied 4**	**Very satisfied 5**
**Vi**	To what extent are you satisfied with divine guidance in your life?	1	2	3	4	5
**Vii**	**How important to you are the aspects of your life covered in questions i-vi in this section?**	**Not at all 1**	**A little 2**	**Moderately 3**	**Very much 4**	**Extremely 5**

**2.1**	**ECOSOCIAL DOMAIN**					
**i**	Activities of daily living[feeding, bathing, toileting, etc]	Fully dependent **1**	Requires substantial help **2**	Requires minimal help **3**	Requires no help but not back to work **4**	Back to work **5**
			**Not at all 1**	**A little 2**	**Moderately 3**	**Very much 4**	**Extremely/ Completely 5**
**ii**	How easy is it for you to communicate with people?	1	2	3	4	5
**iii**	How much support do you get from your relations?	1	2	3	4	5
**iv**	How much respect do you expect from others?	1	2	3	4	5
**v**	How much respect do you get from others?	1	2	3	4	5
**vi**	How much support do you get from your friends?	1	2	3	4	5
**vii**	To what extent are you compelled by others to do what you do not consider suitable for you?	0	1’	2’	3’	4’

©Owolabi MO

**viii**	How well are you able to meet your financial needs?	1	2	3	4	5
**ix**	How surplus is your financial resources?	1	2	3	4	5
**x**	To what extent do you have access to optimal health services?	1	2	3	4	5
**xi**	To what extent do you have access to social support?	1	2	3	4	5
**xii**	How well are you able to manage your home and perform your domestic roles?	1	2	3	4	5
**xiii**	To what extent are you performing your occupational duties?	1	2	3	4	5
**xiv**	How healthy is your physical environment?	1	2	3	4	5
**xv**	To what extent do you have access to transport facilities?	1	2	3	4	5
**xvi**	To what extent do you have opportunities to learn and acquire new skills?	1	2	3	4	5
		**Very dissatisfied 1**	**Dissatisfied 2**	**Neither satisfied nor dissatisfied? 3**	**Satisfied 4**	**Very satisfied 5**
**xvii**	How satisfied are you with your personal relationships?	1	2	3	4	5
**xviii**	How satisfied are you with the support you get from your friends?	1	2	3	4	5
**xix**	How satisfied are you with the conditions of your living place?	1	2	3	4	5
**xx**	How satisfied are you with your access to health services?	1	2	3	4	5
**xxi**	How satisfied are you with your treatment?	1	2	3	4	5
**xxii**	How satisfied are you with your access to transportation?	1	2	3	4	5
**xxiii**	**How important to you are the aspects of your life covered in questions i – xxii in this section?**	**Not at all 1**	**A little 2**	**Moderately 3**	**Very much 4**	**Extremely 5**

©Owolabi MO

2.2	SPIRITUAL INTERACTION DOMAIN	Not at all 1	A little 2	Moderately 3	Very much 4	An extreme amount/ Completely 5
**i**	To what extent do you consider yourself close to God or your object of worship?	1	2	3	4	5
**ii**	To what extent do you meditate and/or study religious books?	1	2	3	4	5
**iii**	To what extent do you discuss aspects of your faith/religion with people of the same religious interest/faith in order to strengthen your individual resolve?	1	2	3	4	5
		**Very dissatisfied 1**	**Dissatisfied 2**	**Neither satisfied nor dissatisfied 3**	**Satisfied 4**	**Very satisfied 5**
**iv**	How satisfied are you with your relationship with God or your object of worship?	1	2	3	4	5
**v**	How satisfied are you with your effort to develop your faith/religion?	1	2	3	4	5
**vi**	**How important to you are the aspects of your life covered in questions i-v above?**	**Not at all 1**	**A little 2**	**Moderately 3**	**Very much 4**	**Extremely 5**

Did someone help you to fill out this form [excluding 1111 i-iii]? **1** interviewer **2** proxy

How long did it take you to fill this form out?

Do you have any comments about this assessment?

Could you please state any other important aspect[s] of your life that has not been assessed.----
