# Supplementary material for: Impact of stroke on health-related quality of life in diverse cultures: the Berlin-Ibadan multicenter international study
Source: Health Qual Life Outcomes. 2011 Sep 27;9:81. doi: 10.1186/1477-7525-9-81 (PMC3206409; doi:10.1186/1477-7525-9-81)
Supplement: Additional file 2 — Health-Related Quality Of Life In Stroke Patients (HRQOLISP) questionnaire-German version. The file contains the complete German version of the HRQOLISP instrument. [file 1477-7525-9-81-S2.PDF]

APPENDIX II : HRQOLISP GERMAN VERSION

INSTRUKTION

Diese Untersuchung fragt Sie wie Sie Ihren gegenwärtigen Zustand von Gesundheit, Lebensqualität und anderen Lebensbereichen wahrnehmen. Bitte beantworten Sie diese Fragen ehrlich. Wenn Sie sich einer bestimmten Antwort unsicher sind, wählen Sie bitte die am ehesten geeignete Antwort aus.

Bitte denken Sie an Ihre Maßstäbe, Hoffnungen, Freuden und Besorgnisse. Denken Sie an Ihr Leben in den letzten zwei Wochen. Kreisen Sie bitte die Nummer ein, die zu Ihrer Antwort am besten passt.

1.1.1.1. : PHYSISCHER ZUSTAND

[i-iii] muss von dem Forscher ausgefüllt werden.

i	Beste motorische Kraft der dominanten oberen Extremität	0 null	1 gering	2 ohne Schwerkraft	3 gegen Schwerkraft	4 gegen Widerstand	5 normal
ii.a	Beste motorische Kraft der betroffenen oberen Extremität	0	1	2	3	4	5
ii.b	Beste motorische Kraft der betroffenen unteren Extremität	0	1	2	3	4	5
iii.	Sprache beeinträchtigt [Aphasie]	Gar nicht 0		Ja 1'			

iv.	**Mobilität**	bettlägerig 1	rollstuhlmobil 2	gehen mit Hilfe 3	gehen mit Hilfsmittel (Stock/ Rollator) 4	gehen ohne Hilfe 5

v	Inwieweit haben Sie Schwierigkeiten (Ihre Hände zu benutzen z. B.) Dinge zu greifen, Türklinken zu drücken, Besteck zu benützen, zu schreiben, Gläser/Dosen zu öffnen, schwere Dinge zu tragen?	**gar nicht 1**	**ein bißchen 2**	**einiger maßen 3**	**sehr 4**	**außerordentlich 5**
vi	Inwieweit haben Sie Schwierigkeiten Ihre Blase/Darm zu kontrollieren?	0	1	2	3	4
vii	Inwieweit haben Sie Schwierigkeiten Sie sich zu setzen/hinzustellen, ohne Ihr Gleichgewicht zu verlieren?	0	1	2	3	4
viii	Inwieweit haben Sie Schwierigkeiten Dinge auf einer Seite zu sehen/ zu erreichen wegen schlechter Sehkraft?	0	1	2	3	4
ix	Inwieweit denken Sie dass physische Schmerzen/Beschwerden/ Unbehangen/abnormales Gefühl/ kein Gefühl Sie daran hindert das zu tun was Sie tun wollen?	0	1	2	3	4
x	Inwieweit brauchen Sie ärztliche Behandlung (Medikamente oder andere Hilfen) und/oder Krankenhausaufenthalte um in Ihrem täglichen Leben zu funktionieren?	0	1	2	3	4
xi	Inwieweit ist Ihr Sexualleben negativ betroffen?	0	1	2	3	4

xii	Wie zufrieden sind Sie mit Ihrer Fähigkeit Tätigkeiten des täglichen Lebens auszuführen (z.B. essen, baden, Körperpflege, Toilettengänge, sich ankleiden, sich zurechtmachen, usw)?	**sehr unzufrieden 1**	**unzufrieden 2**	**weder zufrieden noch unzufrieden 3**	**zufrieden 4**	**sehr zufrieden 5**

xiii	Wie zufrieden sind Sie mit Ihrer Leistungsfähigkeit für Arbeit?	1	2	3	4	5
xiv	Wie zufrieden sind Sie mit Ihrem Sexualleben?	1	2	3	4	5
xv	**Wie wichtig sind für Sie die Aspekte Ihres Lebens, die in diesem Abschnitt (iv-xiv) abgefragt wurden?**	**gar nicht 1**	**ein bisschen 2**	**einiger-maßen 3**	**erheblich/ sehr 4**	**außerordentlich 5**

**1.1.1.2**	**EMOTION/PSYCHE**	**gar nicht/ nie 1**	**ein bißchen /selten 2**	**einiger maßen/ ziemlich oft 3**	**meistens/ sehr oft 4**	**völlig/ immer 5**
**i**	Wie oft haben Sie negative Gefühle z. B. niedergeschlagenes Gefühl, Zorn, Verzweiflung, Sorge, Depression, Angst?	0	1	2	3	4
**ii**	Haben Sie genug Energie für das tägliche Leben?	1	2	3	4	5
**iii**	Inwieweit akzeptieren Sie Ihre körperliche Erscheinung?	1	2	3	4	5
**iv**	Inwieweit haben Sie Freude an Ihrer Arbeit?	1	2	3	4	5
**v**	Wie oft lachen Sie?	1	2	3	4	5
**vi**	Inwieweit genießen Sie Ihre Freizeit, Hobbys, Zeitvertreib, Ruhe, Entspannung?	1	2	3	4	5
**vii**	Wie sicher fühlen Sie sich in Ihrem täglichen Lebens?	1	2	3	4	5
**viii**	Inwieweit haben Sie jemals gedacht dass der Tod besser wäre als Ihr gegenwärtiger Zustand?	0	1	2	3	4
**ix**	Inwieweit haben Sie jemals gedacht Ihr Leben selbst zu beenden?	0	1	2	3	4
**x**	Wie zufrieden sind Sie mit Ihrem Schlaf (Dauer und Qualität) ?	**sehr unzufrieden 1**	**unzufrieden 2**	**weder zufrieden noch unzufrieden 3**	**zufrieden 4**	**sehr zufrieden 5**
**xi**	Wie zufrieden sind Sie mit Ihren Gefühlen?	1	2	3	4	5
**xii**	**Wie wichtig sind für Sie die Aspekte Ihres Lebens, die in diesem Abschnitt (Frage i –xi) abgefragt wurden?**	**gar nicht 1**	**ein bisschen 2**	**einiger maßen 3**	**erheblich/ sehr 4**	**außerordentlich 5**

**1.1.2**	**KOGNITIVER ZUSTAND**	**gar nicht/ nie 1**	**ein bißchen /selten 2**	**einiger maßen/ ziemlich oft 3**	**am meistens/ sehr oft 4**	**völlig/ immer 5**
**i**	Inwieweit können Sie sich konzentrieren?	1	2	3	4	5
**ii**	Inwieweit ist Ihr Gedächtnis beeinträchtigt?	0	1	2	3	4
**iii**	Inwieweit können Sie neue Sachen lernen?	1	2	3	4	5
**iv**	Inwieweit verstehen Sie Ihre Erkrankung?	1	2	3	4	5
**v**	Inwieweit können Sie logische Lösungen zu (Ihre) Problemen planen und Sie sich entscheiden?	1	2	3	4	5

**vi**	Inwieweit können Sie sich entspannen?	1	2	3	4	5
**vii**	Wie verfügbar für Sie ist die Information, die Sie brauchen für Ihr tägliches Leben?	1	2	3	4	5
**viii**	Inwieweit können Sie kommunizieren (verstehen und Sie sich verständigen)?	1	2	3	4	5
**ix**	Wie zufrieden sind Sie mit Ihrem Gedächtnis und Konzentrationsfähigkeit?	**sehr unzufrieden 1**	**unzufrieden 2**	**weder zufrieden noch unzufrieden 3**	**zufrieden 4**	**sehr zufrieden 5**
**x**	Wie zufrieden sind Sie mit Ihrer Kommunikationsfähigkeit?	1	2	3	4	5
**xi**	Wie zufrieden sind Sie mit Ihrer Denk- und Lernfähigkeit?	1	2	3	4	5
**xii**	**Wie wichtig sind für Sie die Aspekte Ihres Lebens, die in diesem Abschnitt (Frage i-xi) abgefragt wurden?**	**gar nicht 1**	**ein bisschen 2**	**einiger maßen 3**	**erheblich/ sehr 4**	**außer-ordentlich 5**

**1.2.1**	**SEELE**	**gar nicht 1**	**ein bißchen 2**	**einiger maßen 3**	**erheblich/ sehr 4**	**außer-ordentlich 5**
**i**	Wie sehr schätzen Sie sich?	1	2	3	4	5
**ii**	Wie viel Selbstvertrauen haben Sie?	1	2	3	4	5
**iii**	Wie viel Vertrauen haben Sie zu Ihrem Gott?	1	2	3	4	5
**iv**	Wie kreativ sind Sie?	1	2	3	4	5
**v**	Inwieweit sind Sie unabhängig und individuell in Ihrem logischem Denken und Entscheidungsfindungen?	1	2	3	4	5
**vi**	Inwieweit glauben Sie dass Ihr Leben einen Zweck hat?	1	2	3	4	5
**vii**	Inwieweit interessieren Sie sich dafür, Ihren Lebenszweck zu erfüllen?	1	2	3	4	5
**viii**	Inwieweit akzeptieren/glauben Sie an Schicksal / Vorherbestimmung?	1	2	3	4	5
**ix**	Inwieweit glauben Sie an Willensfreiheit?	1	2	3	4	5
**x**	Inwieweit denken Sie dass Ihre gegenwärtige Verfassung Sie hindert Ihren Lebenszweck zu erfüllen?	0	1	2	3	4
**xi**	Inwieweit denken Sie dass Ihre gegenwärtige Verfassung Ihnen geholfen hat, Ihren Lebenszweck zu erfüllen?	1	2	3	4	5
**xii**	Inwieweit haben Ihre Träume/Visionen /außersensorische Wahrnehmung (wenn anwesend) Ihre gegenwärtige Verfassung schlecht porträtiert?	0	1	2	3	4
**xiii**	Inwieweit sind Sie intuitive/inspiriert/einfallsreich?	1	2	3	4	5
**xiv**	Inwieweit verlassen Sie sich darauf, dass Gott Ihre Probleme löst?	1	2	3	4	5
**xv**	Inwieweit verlassen Sie sich auf sich selbst Ihre Probleme selber zu lösen?	**gar nicht 1**	**ein bißchen 2**	**einiger maßen 3**	**erheblich /sehr 4**	**außer-ordentlich 5**
**xvi**	Inwieweit glauben Sie daran, dass der Teufel verantwortlich für Ihre gegenwärtige Verfassung ist?	0	1	2	3	4
**xvii**	Inwieweit sind Sie oder andere Leute eher verantwortlich für Ihre gegenwärtige Verfassung als Gott?	0	1	2	3	4
**xviii**	Inwieweit glauben Sie an Leben nach dem Tod?	1	2	3	4	5

**xix**	Inwieweit glauben Sie an Gott?		1	2	3	4	5
**xx**	Inwieweit üben Sie Ihre Religion/Glauben aus?		1	2	3	4	5
**xxi**	Inwieweit akzeptieren Sie Ihre gegenwärtige Verfassung?		1	2	3	4	5
**xxii**	Wie stark ist Ihr Lebenswille?		1	2	3	4	5
**xxiii**	Inwieweit sind sie zufrieden mit Ihrem Glauben an Gott?	**sehr unzufrieden 1**	**unzufrieden 2**	**weder zufrieden noch unzufrieden 3**	**zufrieden 4**	**sehr zufrieden 5**
**xxiv**	Wie zufrieden sind Sie mit sich selbst?	1	2	3	4	5
**xxv**	Wie zufrieden sind Sie mit Ihren Fähigkeiten?	1	2	3	4	5
**xxvi**	**Wie wichtig sind für Sie die Aspekte Ihres Lebens, die in diesem Abschnitt (Frage i-xxv) abgefragt wurden?**	**gar nicht 1**	**ein bisschen 2**	**einiger maßen 3**	**erheblich /sehr 4**	**außer ordentlich 5**

**1.2.2**	**GEIST**	**gar nicht 1**	**ein bisschen 2**	**einiger maßen 3**	**erheblich /sehr 4**	**außer ordentlich 5**
**i**	Inwieweit verstehen Sie Gott?	1	2	3	4	5
**ii**	Inwieweit sind Sie von Gott geleitet/ motiviert in Ihrem (täglichen) Leben?	1	2	3	4	5
**iii**	Inwieweit verstehen Sie Ihre Religion/Glauben?	1	2	3	4	5
**vi**	Inwieweit glauben Sie dass Gott verantwortlich für Ihre gegenwärtige Verfassung ist?	1	2	3	4	5
**v**	Inwieweit empfinden Sie Ihr Leben als sinnvoll?	1	2	3	4	5
**vi**	Inwieweit sind Sie zufrieden mit göttlicher Anleitung in Ihrem Leben?	**sehr unzufrieden 1**	**unzufrieden 2**	**weder zufrieden noch unzufrieden 3**	**zufrieden 4**	**sehr zufrieden 5**
**vii**	**Wie wichtig sind für Sie die Aspekte Ihres Lebens, die in diesem Abschnitt (Frage i-vi) abgefragt wurden?**	**gar nicht 1**	**ein bisschen 2**	**einiger maßen 3**	**erheblich/ sehr 4**	**außer ordentlich 5**

**2.1**	**GESSELLSCHAFTLICHES LEBEN/ SOZIALE INTERAKTION**					
**i**	Tätigkeiten des täglichen Lebens (Essen, Körperpflegen, Toilettengänge, usw)	völlig abhängig **1**	erhebliche Hilfe benötigt **2**	ein bißchen Hilfe benötigt **3**	benötigt keine Hilfe, aber keine Berufstätigkeit **4**	wieder berufstätig **5**
**ii**	Wie einfach ist es für Sie, mit anderen Leuten zu kommunizieren (sich zu unterhalten)	**gar nicht 1**	**ein bisschen 2**	**einiger maßen 3**	**erheblich/ sehr 4**	**außer ordentlich 5**
**iii**	Wie viel Unterstützung bekommen Sie von Ihren Verwandten?	1	2	3	4	5
**iv**	Wie viel Respekt erwarten Sie von anderen Leuten?	1	2	3	4	5
**v**	Wie viel Respekt bekommen Sie von anderen Leuten?	1	2	3	4	5
**vi**	Wie viel Unterstützung bekommen Sie von Ihren Freund/Innen?	1	2	3	4	5

**vii**	Inwieweit werden Sie von anderen Leuten gezwungen etwas zu tun was Sie für sich für ungeeignet halten?	0	1	2	3	4
**viii**	Inwieweit können Sie Ihre finanziellen Bedürfnisse erfüllen?	1	2	3	4	5
**ix**	Wie groß ist Ihre finanzieller Überschuss?	1	2	3	4	5
**x**	Inwieweit sind Ihnen optimale Gesundheitsdienste zugänglich?	1	2	3	4	5
**xi**	Inwieweit ist Ihnen soziale Unterstützung zugänglich?	1	2	3	4	5
**xii**	Inwieweit können Sie Ihren Haushalt führen und Ihre häusliche Rolle spielen?	1	2	3	4	5
**xiii**	Inwieweit kommen Sie Ihren beruflichen Verpflichtungen nach?	1	2	3	4	5
**xiv**	Wie sauber ist Ihre physische Umgebung?	1	2	3	4	5
**xv**	Inwieweit sind Verkehrsmittel für Sie zugänglich?	1	2	3	4	5
**xvi**	Inwieweit haben Sie Gelegenheiten neue Fertigkeiten zu erlernen und anzueignen?	1	2	3	4	5
**xvii**	Wie zufrieden sind Sie mit Ihren persönlichen Beziehungen?	**sehr unzufrieden 1**	**unzufrieden 2**	**weder zufrieden noch unzufrieden 3**	**zufrieden 4**	**sehr zufrieden 5**
**xviii**	Wie zufrieden sind Sie mit der Unterstützung, die Sie von Ihren Freund/Innen bekommen?	1	2	3	4	5
**xix**	Wie zufrieden sind Sie mit Ihrer Wohnsituation?	1	2	3	4	5
**xx**	Wie zufrieden sind Sie mit Ihrem Zugang zu Gesundheitsdiensten?	1	2	3	4	5
**xxi**	Wie zufrieden sind sie mit Ihren Behandlungen?	1	2	3	4	5
**xxii**	Wie zufrieden sind Sie mit Ihrem Zugang zu Verkehrsmitteln?	1	2	3	4	5
**xxiii**	**Wie wichtig sind für Sie die Aspekte Ihres Lebens, die in diesem Abschnitt (Frage i-xxii) abgefragt wurden?**	**gar nicht 1**	**ein bisschen 2**	**einigermaßen 3**	**erheblich/ sehr 4**	**außer-ordentlich 5**

**2.2**	**SPIRITUELLE INTERAKTION**	**gar nicht 1**	**ein bisschen 2**	**einiger maßen 3**	**erheblich/ sehr 4**	**außer-ordentlich 5**
**i**	Inwieweit denken Sie dass Sie Gott (oder Ihrem vergötterten Objekt) nahe stehen?	1	2	3	4	5
**ii**	Inwieweit meditieren Sie und/oder studieren Sie religiöse Bücher?	1	2	3	4	5
**iii**	Inwieweit diskutieren Sie über Aspekte Ihrer Religion/Glaubens mit Leute desselben Glaubens/ Interesse um Ihre individuelle Überzeugung zu stärken?	1	2	3	4	5
**iv**	Wie zufrieden sind Sie mit Ihrer Beziehung zu Gott (oder Ihrem vergötterten Objekt)?	**sehr unzufrieden 1**	**unzufrieden 2**	**weder zufrieden noch unzufrieden 3**	**zufrieden 4**	**sehr zufrieden 5**

**v**	Wie zufrieden sind Sie mit Ihrem Bemühen Ihren Glauben/Religion zu entwickeln?	1	2	3	4	5
**vi**	**Wie wichtig sind für Sie die Aspekte Ihres Lebens, die in diesem Abschnitt (Frage i-v) abgefragt wurden?**	**gar nicht 1**	**ein bisschen 2**	**einiger maßen 3**	**erheblich/ sehr 4**	**außer-ordentlich 5**

Hat jemand Ihnen geholfen diesen Fragenbogen auszufüllen (*ausschließlich 1111i-iii*)
1 Interviewer/in **2** Angehöriger

Wie lange hat es gedauert den Fragenbogen auszufüllen? _________ Minuten

Haben Sie Kommentare zu dieser Untersuchung?

____
____
____
____
____

Würden Sie bitte andere wichtige Aspekte Ihres Lebens nennen, die nicht abgefragt wurden:

____
____
____
____
____
